# Dosimetry of [^18^F]TRACK, the first PET tracer for imaging of TrkB/C receptors in humans

**DOI:** 10.1186/s41181-023-00219-x

**Published:** 2023-10-23

**Authors:** Alexander Thiel, Alexey Kostikov, Hailey Ahn, Youstina Daoud, Jean-Paul Soucy, Stephan Blinder, Carolin Jaworski, Carmen Wängler, Björn Wängler, Freimut Juengling, Shirin A. Enger, Ralf Schirrmacher

**Affiliations:** 1 Jewish General Hospital and Lady Davis Institute for Medical Research, 3755 Chemin de la Cote St. Cathérine, Montreal, Québec H3T 1E2 Canada; 2 Department of Neurology & Neurosurgery, McGill Univesrity, Montreal, Canada; 3 Brain Imaging Center, Montreal Neurological Institute, Montreal, Canada; 4 Department of Chemistry, McGill University, Montreal, Canada; 5 Medical Physics Unit, McGill University, Montreal, Canada; 6 PERFORM Centre Concordia University, Montreal, Canada; 7 Cross Cancer Institute, Medical Isotope Cyclotron Facility, University of Alberta, Edmonton, Canada; 8 Biomedical Chemistry, Clinic of Radiology and Nuclear Medicine, Medical Faculty Mannheim, Heidelberg University, Mannheim, Germany; 9 Molecular Imaging and Radiochemistry, Clinic of Radiology and Nuclear Medicine, Medical Faculty Mannheim, Heidelberg University, Mannheim, Germany; 10 Neuroscience and Mental Health Institute, University of Alberta, Edmonton, Canada; 11 Department of Oncology, Division of Oncologic Imaging, University of Alberta, Edmonton, Canada; 12 Medical Faculty, University Bern, Bern, Switzerland

**Keywords:** Tropomyosin receptor kinases, Positron emission tomography, [^18^F]TRACK, Dosimetry

## Abstract

**Background:**

Reduced expression or impaired signalling of tropomyosin receptor kinases (Trk receptors) are found in a vast spectrum of CNS disorders. [^18^F]TRACK is the first PET radioligand for TrkB/C with proven in vivo brain penetration and on-target specific signal. Here we report dosimetry data for [^18^F]TRACK in healthy humans. 6 healthy participants (age 22–61 y, 3 female) were scanned on a General Electric Discovery PET/CT 690 scanner. [^18^F]TRACK was synthesized with high molar activities (A_m_ = 250 ± 75 GBq/µmol), and a dynamic series of 12 whole-body scans were acquired after injection of 129 to 147 MBq of the tracer. Images were reconstructed with standard corrections using the manufacturer’s OSEM algorithm. Tracer concentration time-activity curves (TACs) were obtained using CT-derived volumes-of-interest. Organ-specific doses and the total effective dose were estimated using the Committee on Medical Internal Radiation Dose equation for adults and tabulated Source tissue values (S values).

**Results:**

Average organ absorbed dose was highest for liver and gall bladder with 6.1E−2 (± 1.06E−2) mGy/MBq and 4.6 (± 1.18E−2) mGy/MBq, respectively. Total detriment weighted effective dose E_DW_ was 1.63E−2 ± 1.68E−3 mSv/MBq. Organ-specific TACs indicated predominantly hepatic tracer elimination.

**Conclusion:**

Total and organ-specific effective doses for [^18^F]TRACK are low and the dosimetry profile is similar to other ^18^F-labelled radio tracers currently used in clinical settings.

## Introduction

Trk receptors in the central nervous system (CNS) regulate many aspects of neuronal development and function, such as cell differentiation, dendritic outgrowth, and synaptic plasticity. They are classified according to the neurotrophins they interact with (Reichardt 2006): TrkA is activated through nerve growth factor (NGF), TrkB interacts with brain-derived neurotrophic factor (BDNF) and neurotrophin-4 (NT4) while neurotrophin 3 (NT-3) binds to TrkC.

Within the CNS, reduced expression or abnormal and impaired signalling of Trk receptors are found in a vast spectrum of disorders and pathologies, such as ischemic stroke, Alzheimer’s disease (AD) and others (Tejeda and Díaz-Guerra 2017).

We developed [^18^F]TRACK, an ^18^F-derivative of [^11^C]-(*R*)-IPMICF16 (Bernard-Gauthier et al. 2017), displaying significantly reduced P-gp-liability, brain off-target selectivity, and favorably adjusted binding affinity in TrkB/C rich regions (Bernard-Gauthier et al. 2018). [^18^F]TRACK showed excellent in vivo pharmacokinetic properties in all species including humans (Bailey et al. 2019), permeates the blood–brain-barrier rapidly with fast reversible binding kinetics and a high specific signal as demonstrated by competition studies (Bernard-Gauthier et al. 2017). Finally, in line with previous immunohistochemical detection and in situ hybridization studies of post-mortem tissue, this tracer adequately quantified the TrkB/C density reduction in the hippocampus of AD patients as compared to healthy brains in initial in vitro studies (Bernard-Gauthier et al. 2017, 2018). The objective of this study was to provide dosimetry data for [^18^F]TRACK in humans.

## Methods

We studied 6 healthy participants (3 male, 3 female) between 22 and 61 years of age with no history of metabolic, gastrointestinal, cardiovascular or psychiatric disorders. Subjects were recruited through advertisements following ethics board approval (CCER20-21–03). Demographic data, injected doses and injected masses are summarized in Table 1.

**Table 1:** Participant demographics, molar [^18^F]TRACK activity (A_m_) and injected activity and mass

Subject	Sex	Age	Body weight [kg]	A_m_ [GBq/µmol]	Injected activity [MBq]	Injected mass [µg]
S1	Male	26	85	389	133.3	0.323
S2	Female	59	49	236	129.3	0.376
S3	Female	24	60	250	156.4	0.367
S4	Female	54	65	144	129.0	0.663
S5	Male	42	69	277	138.4	0.328
S6	Male	18	74	203	147.0	0.411
Mean ± SD				250 ± 82	138.9 ± 10.90	0.41 ± 0.13

[^18^F]TRACK was synthesized as previously described (Mossine et al. 2017). Radiochemical yield was 4.4 ± 0.8% not corrected for decay (activity range 3.5–5.7 GBq) with > 99% radiochemical purity and molar activities of 250 ± 82 GBq/µmol at the end of synthesis. No effect of radiotracer on vital signs was recorded during the scan.

PET scans were performed on a General Electric Discovery PET-CT 690 scanner (GE Healthcare, Milwaukee, WI) at the PERFORM Centre, Concordia University, Montréal. First, whole body CT scans were obtained for attenuation correction and anatomical localization. Subsequently, an intravenous bolus of [^18^F]TRACK was injected over 1 min (dose range: 129 to 147 MBq) followed by 12 whole body emission scans of 8 bed positions each.

PET images were reconstructed with all standard corrections using the proprietary Ordered Subset Expectation Maximization (OSEM) algorithm provided by the camera’s manufacturer, resulting in a dynamic series of 12-time frames sampled on a 256 × 256 × 299 matrix with a reconstructed voxel size of 2.73 × 2.73 × 3.27 mm^3^.

A set of 13 source organs and one region representing the rest of the body (Table 2) were segmented on each participant’s CT scan using the Velocity Software (https://www.varian.com/products/interventional-solutions/velocity) from which binary 3D masks for each organ were created. The activity in Bq/mL from each voxel of each frame was obtained, activity values were multiplied by voxel volume to obtain total activity in MBq per organ and time integrated activity (TIA) was calculated for each voxel. For each organ, the TIA 3D matrix was convoluted to the corresponding mask to only extract the cumulated activity for the respective organ, from which the average cumulated activity was computed and normalized to injected activity to obtain time integrated activity coefficients (TIAC).

**Table 2:** Source organs, peak activity and peak time

Source organ	First peak activity [MBq]	First peak time [min]	Second peak activity [MBq]	Second peak time [min]
Urinary bladder	0.07	2	0.138	95
Bone/ bone marrow	13.0	4.5	–	–
Brain	2.4	4.5	–	–
Gall bladder	0.55	4.5	0.94	85
Heart	8.84	2	–	–
Kidney	5.67	2	–	–
Colon content	0.55	2	0.60	397
Liver	37.00	9	–	–
Lungs	27.3	2	–	–
Ovaries	0.02	2	–	–
Small bowel	1.28	2	2.48	397
Testes	0.07	2	–	–
Thyroid	0.14	2	–	–
Rest of body	70	2	–	–

The radiation doses absorbed by the target organs from surrounding source organs were calculated using the MIRD method (Cherry et al. 2012). The absorbed target organ dose per unit activity is the sum of the cumulated activity in each of the source organs multiplied by a dose factor (S-value) in units of Gy/Bq*s (MIRD-Calc software V1.1-Genesis (Carter et al. 2021; Kesner et al. 2018)). The effective (E) and detriment weighted dose (E_DW_) for each subject were obtained by summation of the equivalent dose multiplied by the organ specific weighting factors.

## Results

**Fig. 1:**
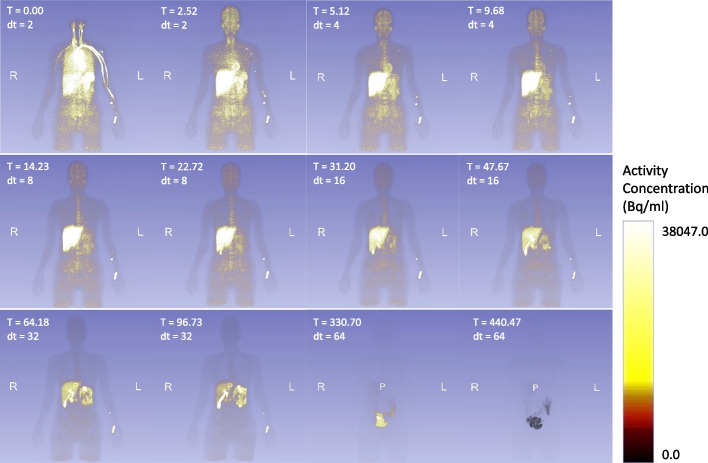
Example of radiotracer distribution in the body over time (maximum intensity projection for each time frame, *T* Frame start time in minutes, *dt* Frame duration in minutes)

**Fig. 2:**
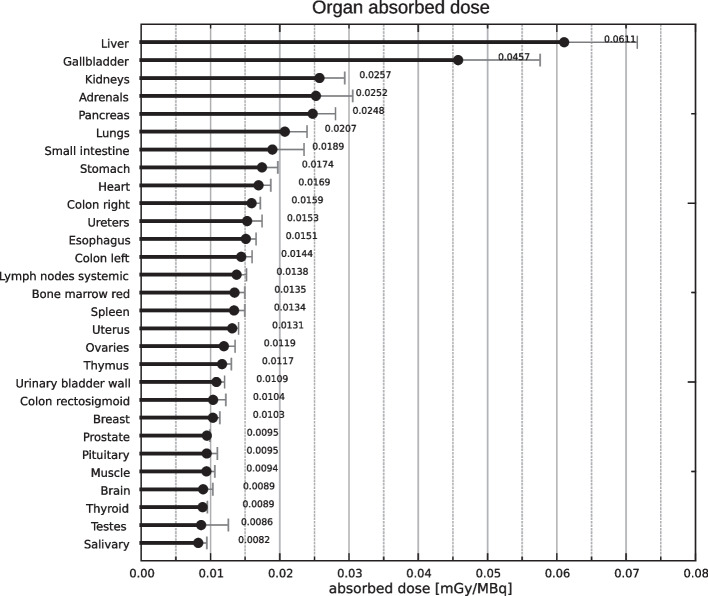
Organ specific mean absorbed doses and standard deviation (*N* = 3 for reproductive organs, *N* = 6 all other organs and tissues)

The distribution of radioactivity over time is illustrated for one subject (Fig. 1); peak activities for all source organs are listed in Table 2. Average time activity curves for all source organs are shown in the Fig. 2. All source organs show a first activity peak within minutes after tracer injection related to perfusion and rapid organ uptake (e.g., liver). Highest activities were found in liver, lungs, bone, kidneys and brain. Organs of the digestive tract (gall bladder, small bowel, and colon) also showed a delayed second activity peak and larger inter-individual variability consistent with hepato-biliary elimination and variations in intestinal motility (Fig. 2).

**Fig. 3:**
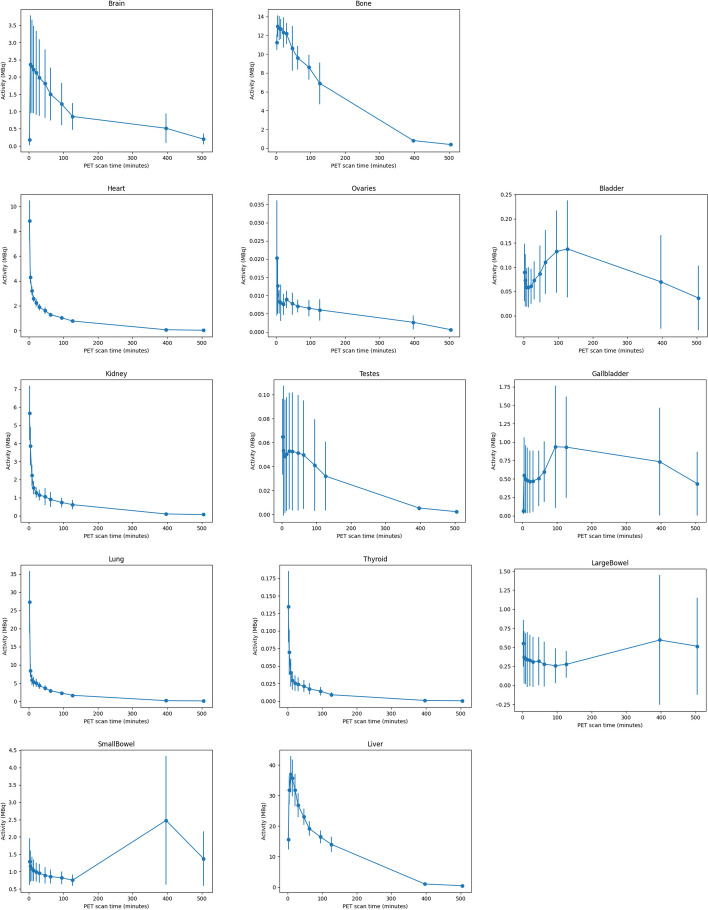
Average time activity curves for all source organs

The model explained on average 89.7 (± 3.27) % of the total injected activity (Table 3). Consistent with hepato-biliary elimination, the absorbed dose coefficients were highest for liver and gall bladder with 6.1E−2 (± 1.06E−2) mGy/MBq and 4.6 (± 1.18E−2) mGy/MBq respectively. Absorbed dose coefficients for reproductive organs were low with 1.12E−2 (± 0.16E−2) mGy/MBq for the ovaries and 0.9E−2 (± 0.39E−2) mGy/MBq for the testicles. Absorbed dose coefficients for all target organs are listed in Fig. 3.

**Table 3:** Detriment weighted (E_DW_) and effective (E) dose coefficients

Subject	E_DW_ [mSv/MBq]	σ E_DW_	E [mSv/MBq]	σ E	% Activity accounted for	Injeccted Activity [MBq]	Projected E_DW−inj_ [mSv]	σ E_DW−inj_	Projected E_DW−370_ [mSv]	σ E_DW−370_
S1	0.0154	4.87E−4	0.0170	5.41E−4	91	133	2.26	0.072	6.28	0.200
S2	0.0192	6.12E−4	0.0175	5.57E−4	94	129	2.26	0.072	6.48	0.206
S3	0.0161	4.92E−4	0.0149	4.49E−4	85	156	2.32	0.070	5.51	0.166
S4	0.0177	5.40E−4	0.0164	4.94E−4	92	129	2.12	0.064	6.07	0.183
S5	0.0147	4.54E−4	0.0158	4.95E−4	88	138	2.18	0.068	5.85	0.183
S6	0.0145	3.98E−4	0.0157	4.37E−4	88	147	2.31	0.064	5.81	0.162
Mean and SD	0.0163 ± 1.84E-3		0.0162 ± 0.86E−3		89.7 ± 3.27	138.7 ± 10.86	2.24 ± 0.07		6.00 ± 0.35	

E_DW−inj_ are the estimated detriment weighted doses for each subject’s injected activity, E_DW-370_ is an estimated dose for a hypothetical injected activity of 370 MBq

The equivalent dose coefficients ranged from 1.49 to 1.70 mSv/MBq with an average of 1.62E−2 (± 0.86E−3) mSv/MBq. Range for the detriment weighted dose (E_DW_) coefficient was similar from 1.45 to 1.92 mSv/MBq with an average E_DW_ across subjects of 1.63E−2 (± 1.68E−3). This corresponded to an E_DW_ of 2.12 to 2.32 mSv for the injected doses, or 5.51–6.48 mSv for a hypothetical dose of 370 MBq (Table 3).

## Discussion

This is the first report on biodistribution and dosimetry of the new TrkB/C receptor ligand [^18^F]TRACK in healthy humans.

The average equivalent dose coefficient estimate was 16.3 µSv/MBq or 6.00 mSv for a standard injected dose of 370 MBq. Following injection, [^18^F]TRACK is rapidly distributed throughout the body with rapid uptake in liver, lungs, kidneys and brain. Consistent with the mainly hepatobiliary elimination, organ absorbed doses were high in the liver, gallbladder, and kidneys.

The equivalent dose coefficient estimates are well within the range of those for other ^18^F-labelled radiotracers (Jackson et al. 2020) such as [^18^F]FDG (19 µSv/MBq) or [^18^F]DOPA (26 µSv/MBq) (Kaushik et al. 2013), and those ^18^F-labelled tracers with primarily hepatobiliary elimination like Florbetapir (18.6 µSv/MBq) (Joshi et al. 2014). With appropriate adjustment of the injected dose, these dosimetry results will allow for repeated measurements in the same subjects without exceeding dose limitations in most jurisdictions (Jackson et al. 2020), which may be of interest when studying TrkB/C receptor density in neurodegeneration or during recovery from focal brain injury. The dosimetry model used in this study (Carter et al. 2021; Kesner et al. 2018) on average accounted for 89.7% of injected activity with the residual activity accumulated below the knees outside the field of view and to some extent at the wall of the injection tubing due to lipophilicity of the tracer.

## Conclusion

In conclusion, [^18^F]TRACK, the first radiotracer for in vivo imaging of TrkB/C receptor density has a dosimetry profile that is similar to other ^18^F-labelled radiotracers currently used in clinical settings.

## Data Availability

The datasets generated during and/or analysed during the current study are available from the corresponding author on reasonable request.

## Abbreviations

Trk: Tropomyosin receptor kinases
A_m_: Molar activity
TAC: Time-activity curve
MIRD: Medical Internal Radiation Dose
CNS: Central nervous system
NGF: Nerve growth factor
BDNF: With brain-derived neurotrophic factor
NT4: Neurotrophin-4
NT-3: Neurotrophin 3
AD: Alzheimer’s disease
OSEM: Ordered Subset Expectation Maximization
TIA: Time integrated activity
TIAC: Time integrated activity coefficients
E: Effective dose
E_DW_: Detriment weighted dose

## References

[CR1] Bailey JJ, Kaiser L, Lindner S, Wust M, Thiel A, Soucy JP (2019). First-in-human brain imaging of [(18)F]TRACK, a PET tracer for tropomyosin receptor kinases. ACS Chem Neurosci.

[CR2] Bernard-Gauthier V, Bailey JJ, Mossine AV, Lindner S, Vomacka L, Aliaga A (2017). A kinome-wide selective radiolabeled TrkB/C inhibitor for in vitro and in vivo neuroimaging: synthesis, preclinical evaluation, and first-in-human. J Med Chem.

[CR3] Bernard-Gauthier V, Mossine AV, Mahringer A, Aliaga A, Bailey JJ, Shao X (2018). Identification of [(18)F]TRACK, a fluorine-18-labeled tropomyosin receptor kinase (Trk) inhibitor for PET imaging. J Med Chem.

[CR4] Carter L, Ocampo Ramos J, Zanzonico P, Bolch W, Kesner A (2021). Comparative evaluation of the new MIRDcalc dosimetry software across a compendium of radiopharmaceuticals. J Nucl Med.

[CR5] Cherry SR, Sorenson JA, Phelps ME, Cherry SR, Sorenson JA, Phelps ME (2012). chapter 22 - internal radiation dosimetry. Physics in nuclear medicine.

[CR6] Jackson IM, Lee SJ, Sowa AR, Rodnick ME, Bruton L, Clark M (2020). Use of 55 PET radiotracers under approval of a radioactive drug research committee (RDRC). EJNMMI Radiopharm Chem.

[CR7] Joshi AD, Pontecorvo MJ, Adler L, Stabin MG, Skovronsky DM, Carpenter AP (2014). Radiation dosimetry of florbetapir F 18. EJNMMI Res.

[CR8] Kaushik A, Jaimini A, Tripathi M, D'Souza M, Sharma R, Mishra AK (2013). Estimation of patient dose in (18)F-FDG and (18)F-FDOPA PET/CT examinations. J Cancer Res Ther.

[CR9] Kesner A, Olguin E, Zanzonico P, Bolch W (2018). MIRDCalc V 1.0 - a community spreadsheet tool for organ-level radiopharmaceutical absorbed dose calculations. J Nucl Med.

[CR10] Mossine AV, Brooks AF, Bernard-Gauthier V, Bailey JJ, Ichiishi N, Schirrmacher R (2017). Automated synthesis of PET radiotracers by copper-mediated 18F-fluorination of organoborons: importance of the order of addition and competing protodeborylation. J Label Compd Radiopharm.

[CR11] Reichardt LF (2006). Neurotrophin-regulated signalling pathways. Philos Trans R Soc B: Biol Sci.

[CR12] Tejeda GS, Díaz-Guerra M (2017). Integral characterization of defective BDNF/TrkB signalling in neurological and psychiatric disorders leads the way to new therapies. Int J Mol Sci.

